# Supplementary material for: ISCA1 is essential for mitochondrial Fe4S4 biogenesis in vivo
Source: Nat Commun. 2017 May 11;8:15124. doi: 10.1038/ncomms15124 (PMC5437272; doi:10.1038/ncomms15124)
Supplement: Supplementary Information — Supplementary Figures, Supplementary Tables. [file ncomms15124-s1.pdf]

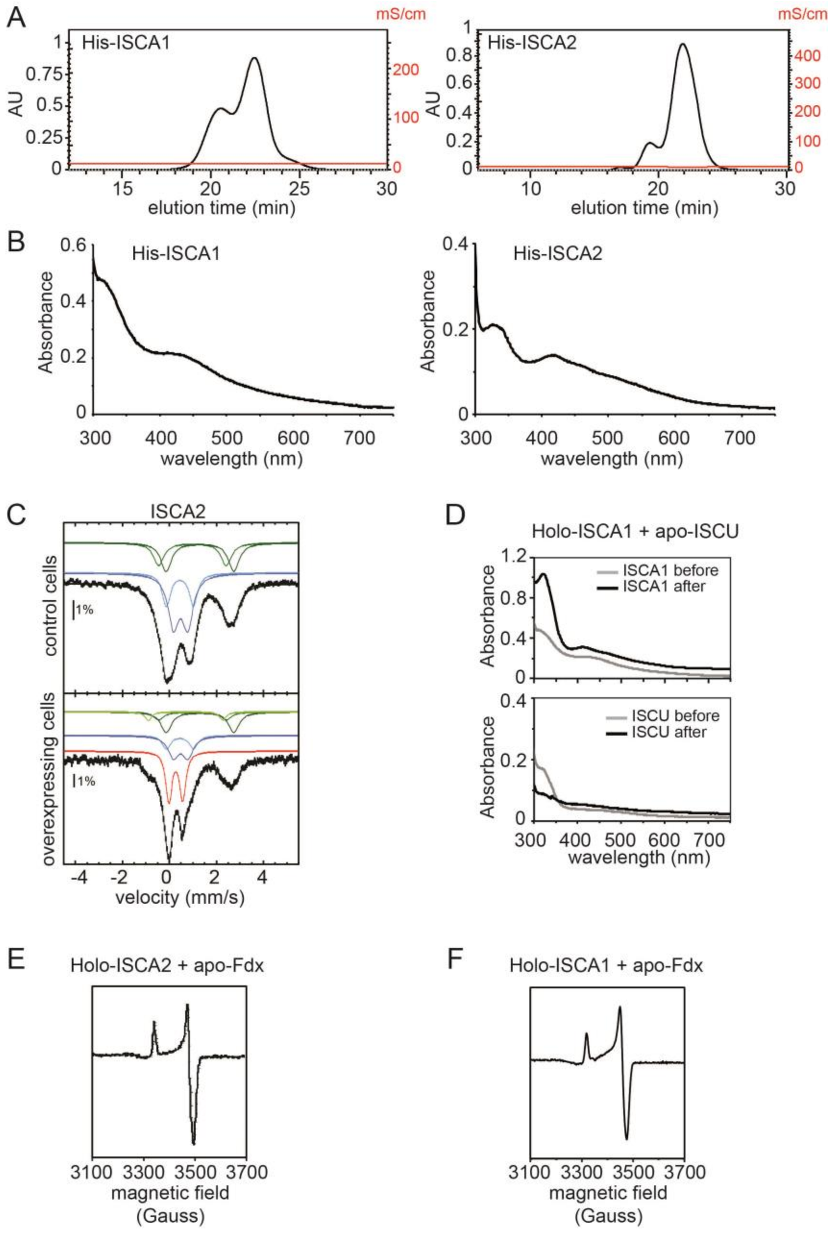

Supplementary Figure 1 Characterization of recombinant ISCA1 and ISCA2 proteins. (A) Elution profiles obtained for ISCA1 and ISCA2 by gel filtration on analytical Superdex-75 equilibrated with buffer H (0.1 M Tris-HCl pH 8, 150 mM NaCl). Flow rate: 0.5 mL/min. ISCA1 elutes in two peaks at 20.29 min and 22.26 min whereas ISCA2 elutes mainly at 21.55 min. AU: Arbitrary unit; mS/cm: conductivity in millisiemens per centimeter. (B) UV-visible spectra of the as-isolated ISCA1 and ISCA2 in buffer I (0.1 Tris-HCl pH 8, 50 mM NaCl). (C) Mössbauer spectra recorded on whole control cells (upper panel) and ISCA2-overexpressing cells (lower panel) at 5.5 K using a 60 mT external magnetic field applied parallel to the γ-beam. Experimental spectra are shown with hatched marks and simulations are overlaid as solid black lines. Up to six components were used for simulation: HS Fe^{II} (green traces), Fe_4S_4 clusters and LS ferrous hemes (light blue trace), Fe^{III} NP (dark blue trace), and $[Fe_2S_2]^{2+}$ (red trace). Simulations were performed as for spectra shown in Figure 1C and parameters are listed in Supplemental Table 1. The nuclear parameters of the HS Fe^{II} slightly differ from those obtained upon the pET plasmid. Diferric $[Fe_2S_2]^{2+}$ clusters are detected in the induced ISCA2 cell sample that indeed account for 30% of the total iron content. A small amount

(6% of the total iron content) of a third high-spin ferrous species (the lightest green line) is also observed in the ISCA2 sample. It is very similar to that detected upon Fe-S cluster assembly on ^{Nif}IscA (see ref 56 in the main text, Mapolelo et al. *Biochemistry*, **51**, 8071 (2012)). (D) UV-visible spectra obtained before and after incubation and separation on Superdex-75 of Fe_2S_2-ISCA1 and apo-ISCU. (E) X-band EPR spectrum of the dithionite (1 mM) reduced ferredoxin (100 μM) after incubation with Fe_2S_2-ISCA2 for 30 min and separation. Temp: 10 K, microwave power: 25 μW, gain: 2.10^4, modulation: 10 gauss. (F) X-band EPR spectrum of the dithionite (1 mM) reduced ferredoxin (100 μM) after incubation with Fe_2S_2-ISCA1 for 30 min and separation. Temp: 10 K, microwave power: 25 μW, gain: 2.10^4, modulation: 10 Gauss.

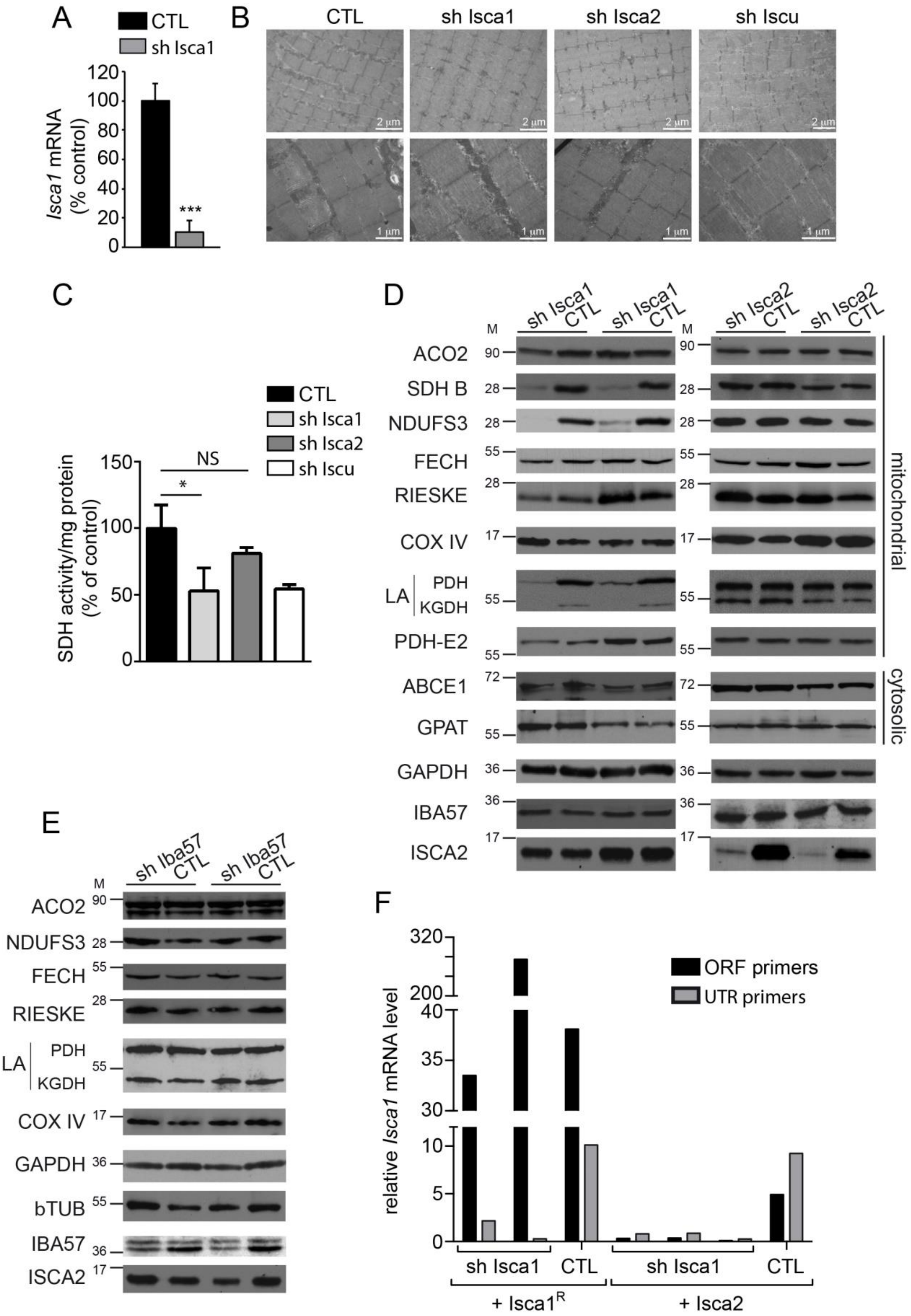
A
CTL
sh Isca1
Isca1 mRNA (% control)
120
100
80
60
40
20
0
***
B
CTL
sh Isca1
sh Isca2
sh Iscu
2 μm
1 μm
C
CTL
sh Isca1
sh Isca2
sh Iscu
SDH activity/mg protein (% of control)
150
100
50
0
NS
*
D
sh Isca1
CTL
sh Isca2
M
ACO2
SDH B
NDUFS3
FECH
RIESKE
COX IV
LA
PDH
KGDH
PDH-E2
ABCE1
GPAT
GAPDH
IBA57
ISCA2
90
28
55
17
72
36
mitochondrial
cytosolic
E
sh Iba57
CTL
M
ACO2
NDUFS3
FECH
RIESKE
LA
PDH
KGDH
COX IV
GAPDH
bTUB
IBA57
ISCA2
F
ORF primers
UTR primers
relative Isca1 mRNA level
320
200
40
35
30
10
5
0
sh Isca1
CTL
+ Isca1R
+ Isca2

Supplementary Figure 2 Knockdown experiments in skeletal muscle. (A) *Isca1* mRNA expression 3 wpi in TA muscle injected with rAAV-shISCA1 (n=5) or rAAV-scrambled shRNA (n=4) (CTL). Results are given as the mean ± SD. ***p<0.0001. (B) TA muscle structure observed by electron microscopy 3 weeks after injection with rAAV-scrambled shRNA (CTL), rAAV-shIsca1, rAAV-shIsca2 or rAAV-shIscu. (C) Succinate dehydrogenase activity measured from TA muscle at 3 wpi, injected with rAAV-scrambled shRNA, rAAV-shIsca1, rAAV-shIsca2 or rAAV-shIscu (positive control). n=3 except for shIscu n=2 (D) Representative western blots for the indicated proteins using extracts from TA muscle at 6 wpi, injected with rAAV-scrambled shRNA (CTL), rAAV-shIsca1 or rAAV-shIsca2. GAPDH was used as loading control (n=3)(E) Representative western blots for the indicated proteins using extracts from TA muscle at 6 wpi, injected with rAAV-scrambled shRNA (CTL) or rAAV-shIba57. GAPDH and beta-tubuline (bTUB) were used as loading controls. (F) Relative *Isca1* mRNA expression 3 wpi in TA muscle co-injected with rAAV-shISCA1 or rAAV-scramble shRNA (CTL) and rAAV-ISCA1^{R} or rAAV-ISCA2 as indicated. ORF primers were used to quantify exogenous + endogenous expression, whereas UTR primers were used to quantify endogenous *Isca1* expression only.

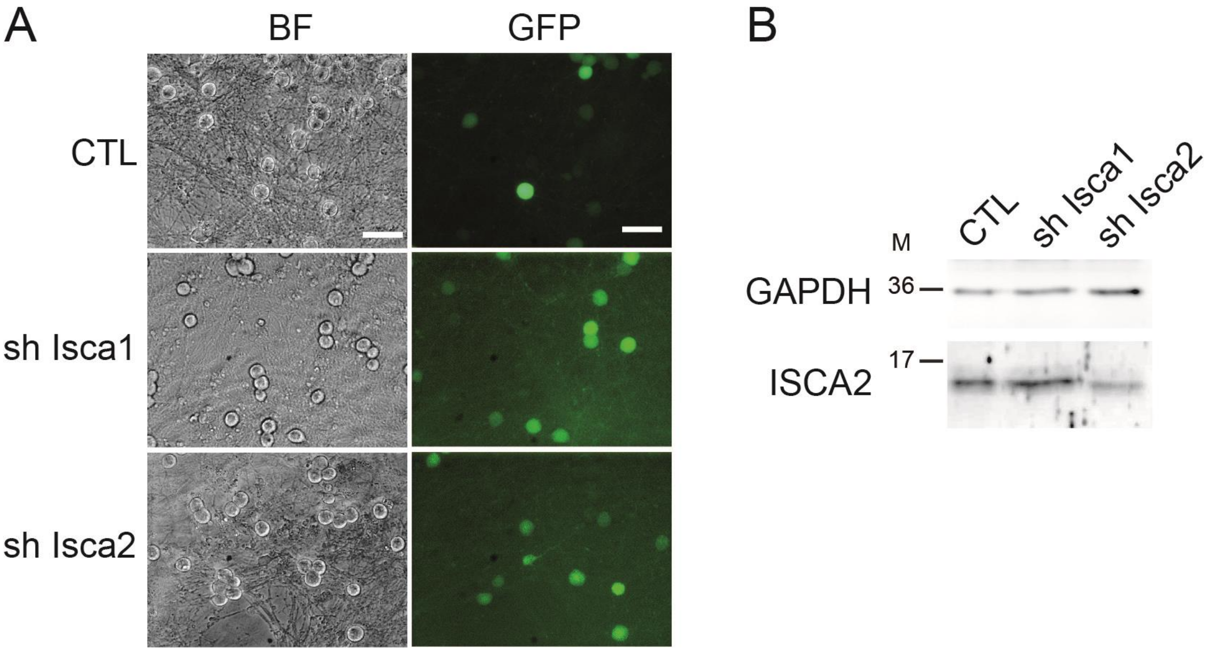

Supplementary Figure 3 Infection of sensory neurons in XF96 microplates. (A) Representative pictures of sensory neurons in XF96 microplate 4 days post-infection, observed by bright field (BF) or by looking at GFP-positive cells by immunofluorescence (GFP). Scale bar = 100 µm (B) Western blots for ISCA2 obtained after Seahorse® measurement and after pooling the wells infected with rAAV-scrambled shRNA (CTL), rAAV-shIsca1 or rAAV-shIsca2. GAPDH was used as loading control.

Original blots for Figure 3:
ISCA1 IP (3 blots, different exposures)

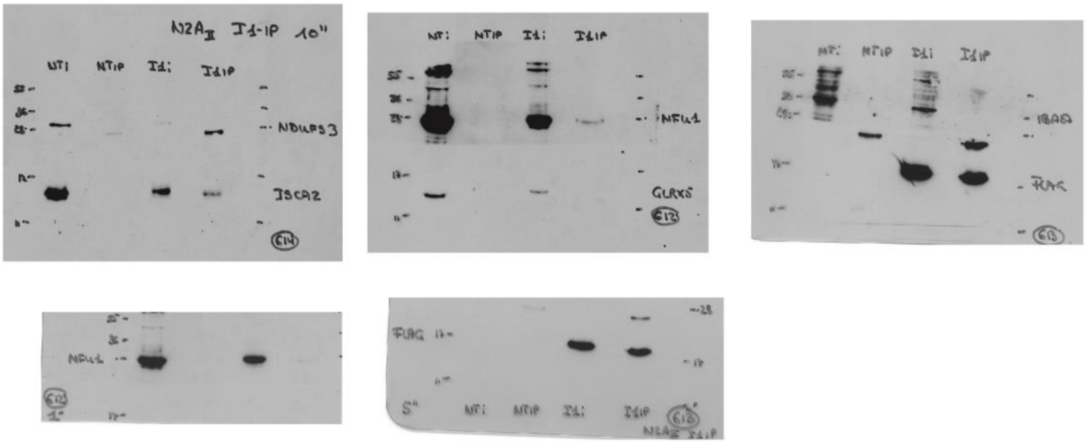

Original blots for Figure 3:
ISCA2 IP (2 blots, different exposures)

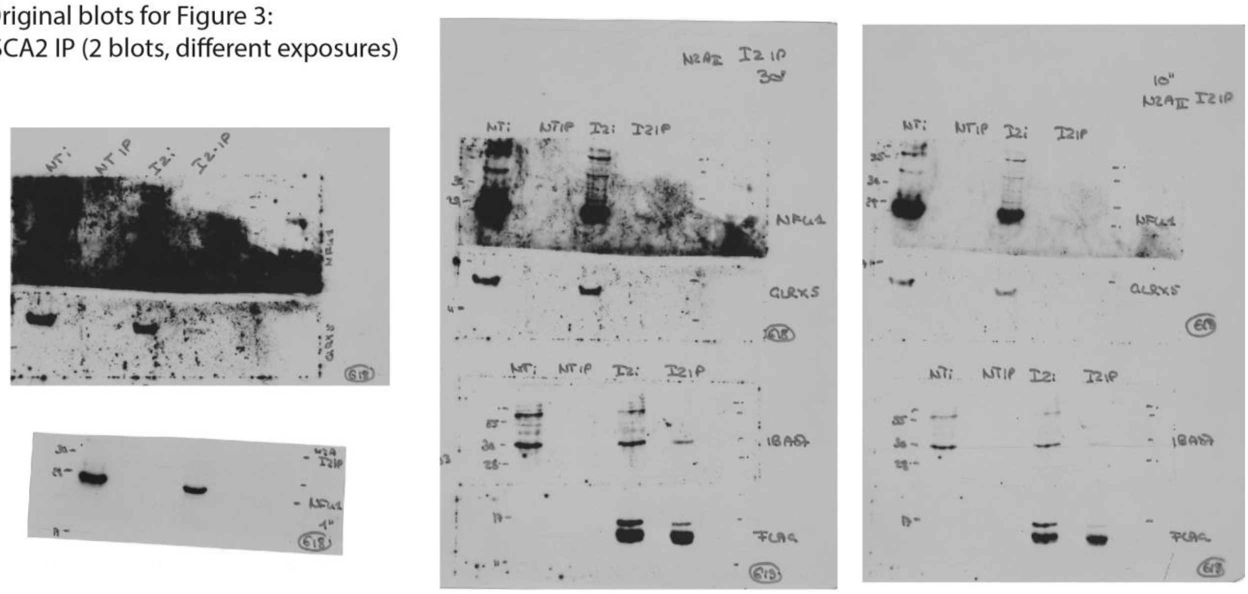

Original blots for Figure 3:
IBA57 IP (2 blots, different exposures)

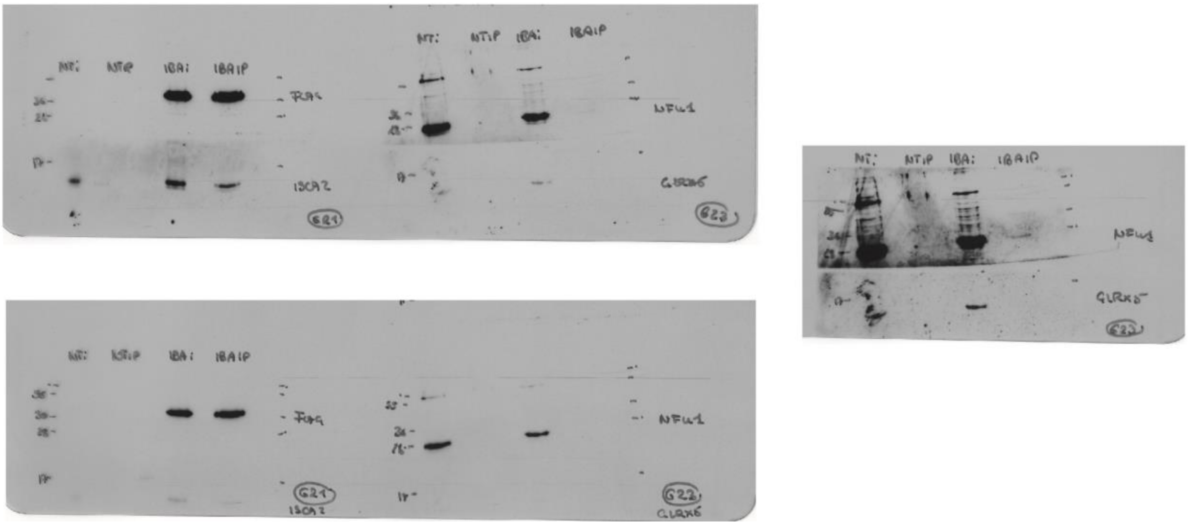

Original blots for Figure 4A:
ISCA1 knockdown

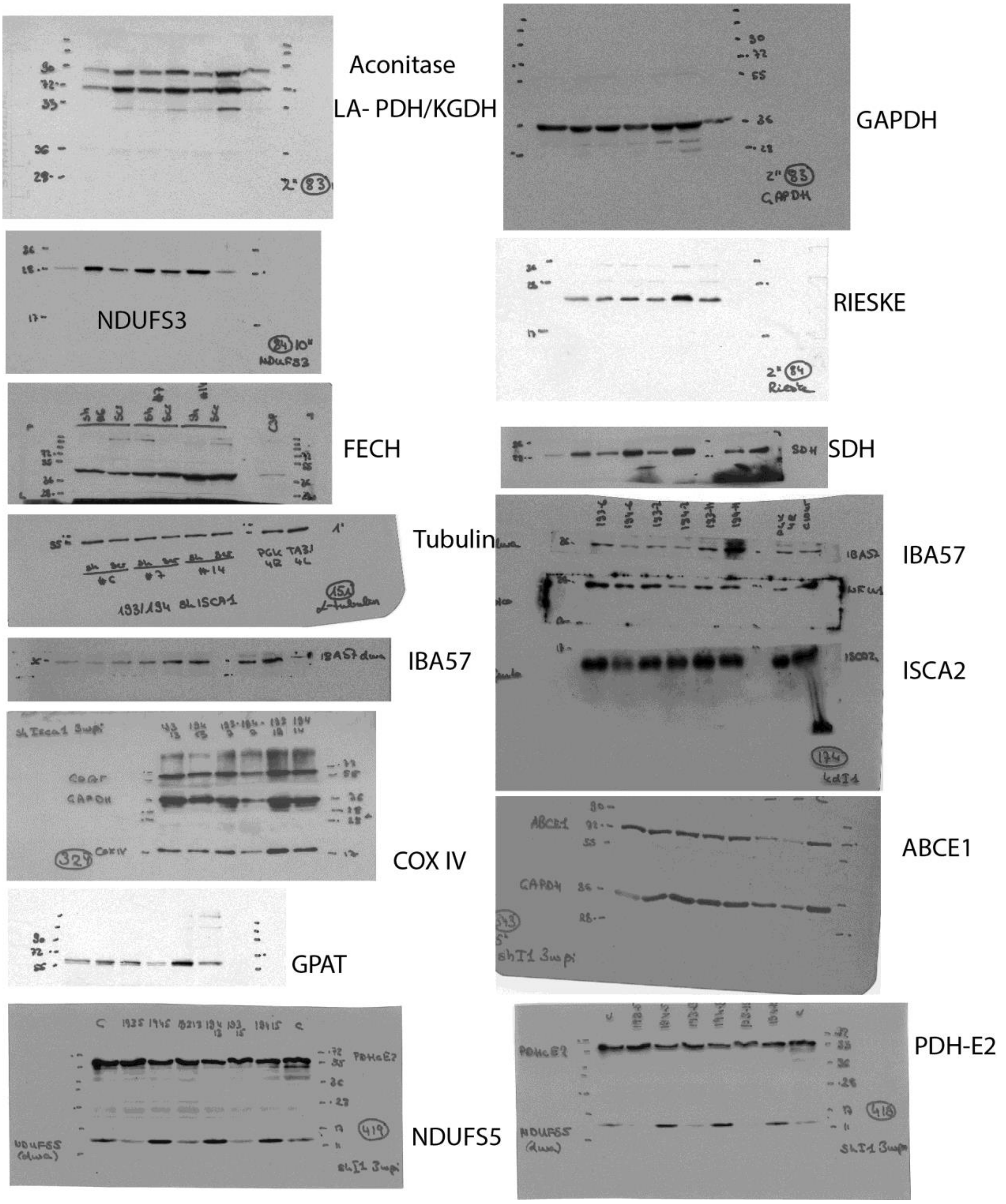

Original blots for Figure 4A:
ISCA2 knockdown

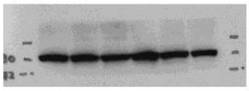

Aconitase

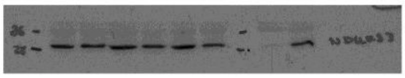

NDUFS3

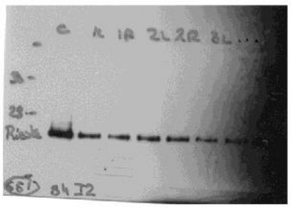

RIESKE

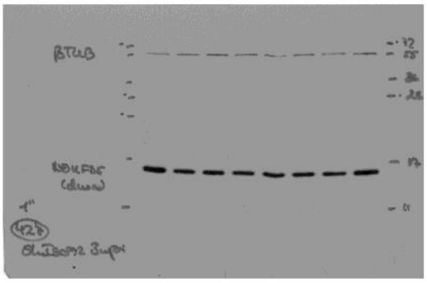

Tubulin

NDUFS5

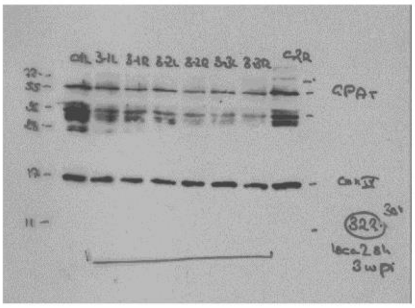

COX IV

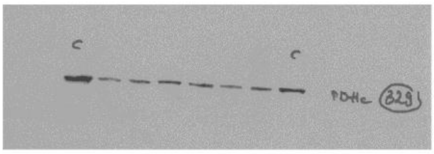

PDH-E2

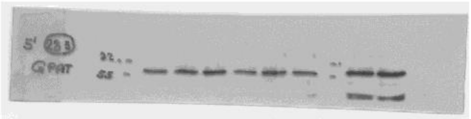

GPAT

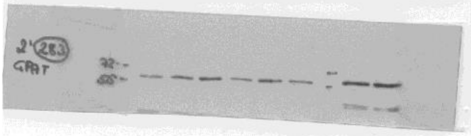

GPAT

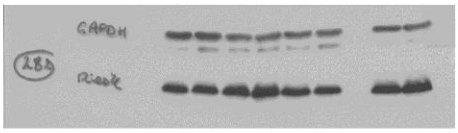

GAPDH

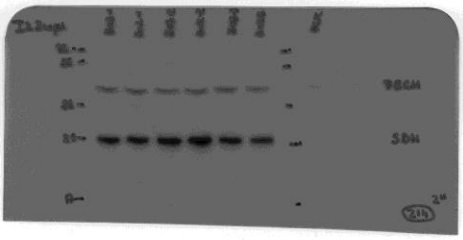

FECH

SDH

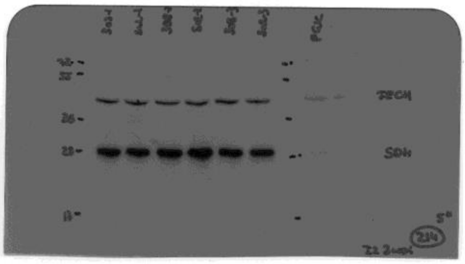

FECH

SDH

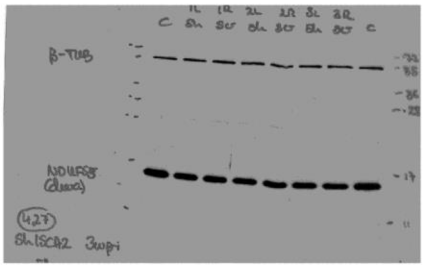

Tubulin

NDUFS5

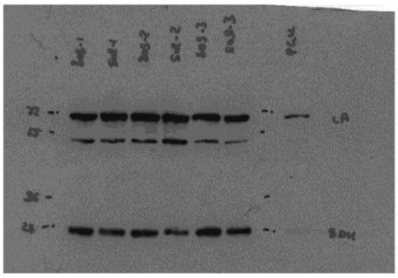

LA- PDH/KGDH

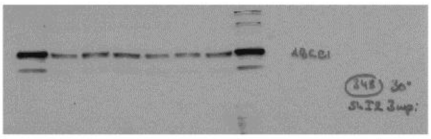

ABCE1

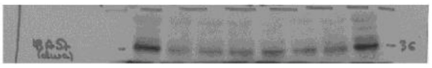

IBA57

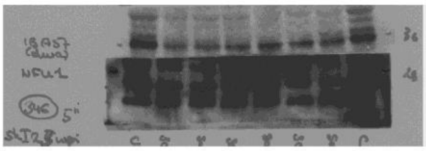

IBA57

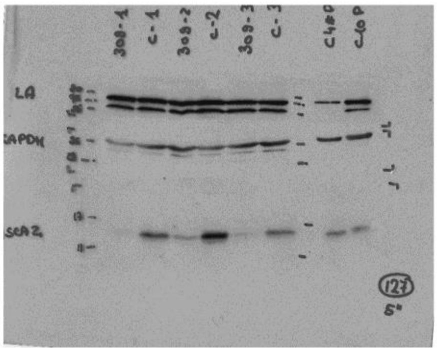

ISCA2

Original blots for Figure 4A:
ISCU knockdown

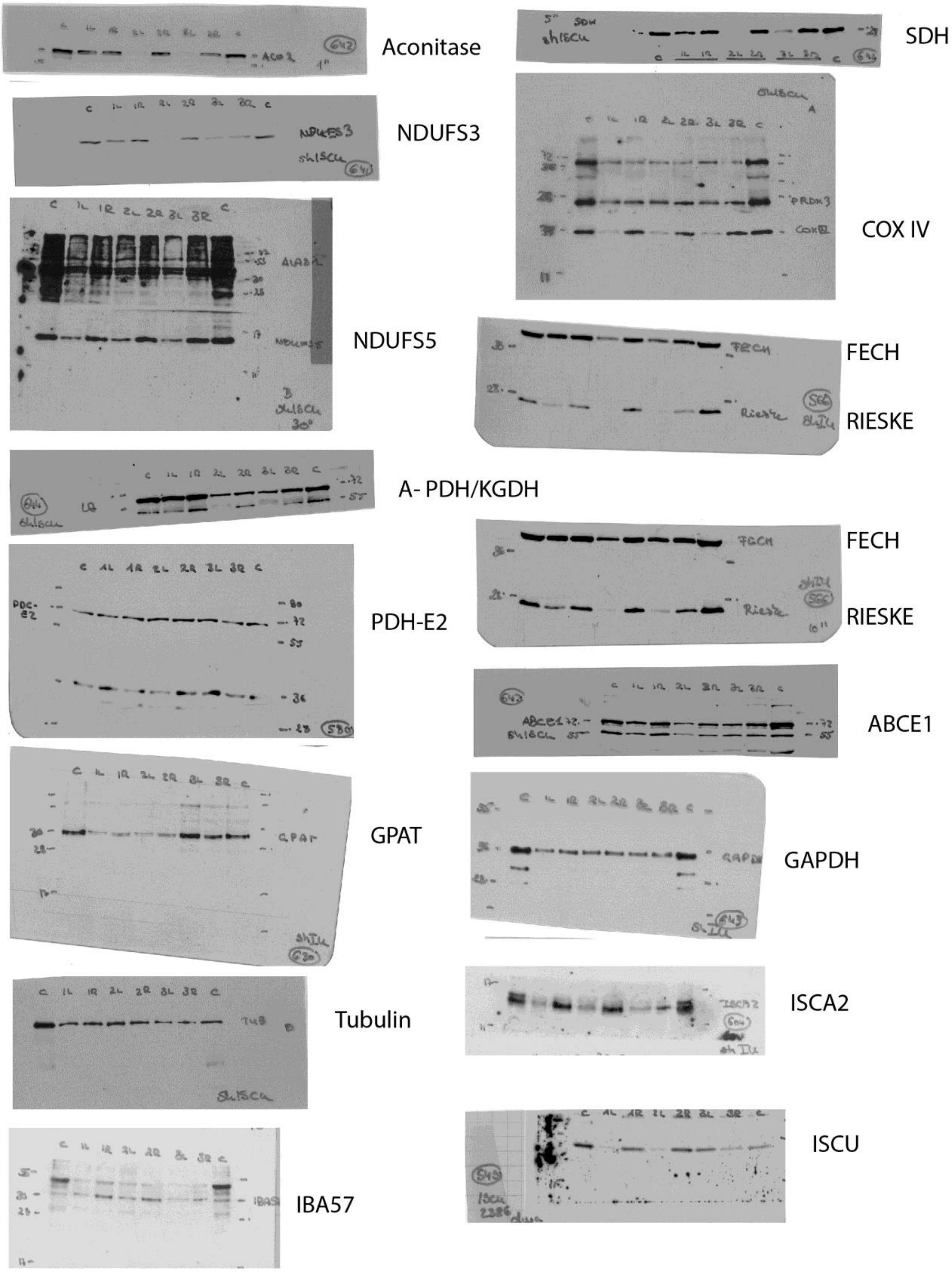

Original blots for Figure 4C:
ISCA1 knockdown, ISCA1R

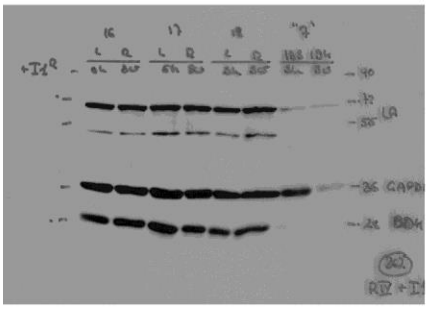

LA- PDH/KGDH

GAPDH

SDH

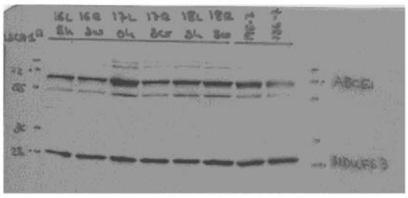

NDUFS3

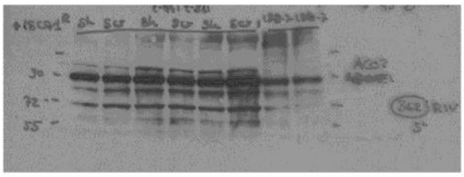

Aconitase

ISCA1 knockdown, ISCA2R

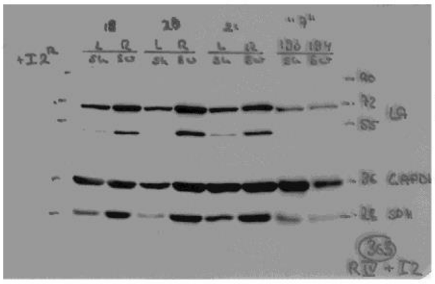

LA- PDH/KGDH

GAPDH

SDH

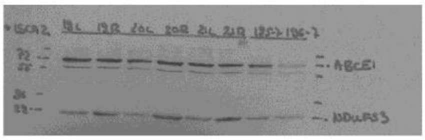

NDUFS3

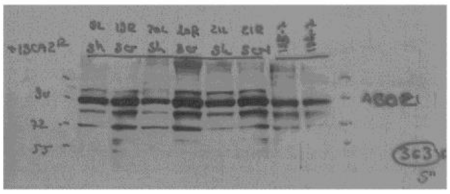

Aconitase

ISCA1 knockdown, ISCA1R
ISCA1 knockdown, ISCA1R
ISCA1 knockdown, ISCA2R
ISCA1 knockdown, ISCA2R

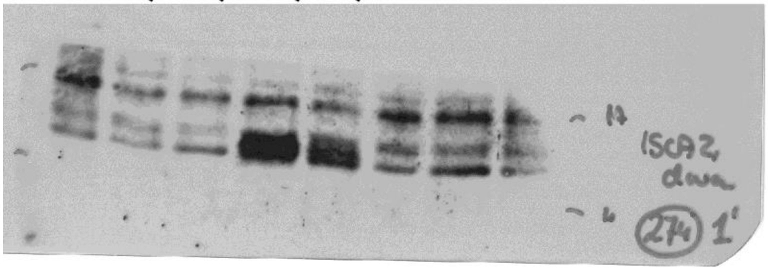

ISCA2

Original blots for Figure S2D:
ISCA1 knockdown 6 wpi

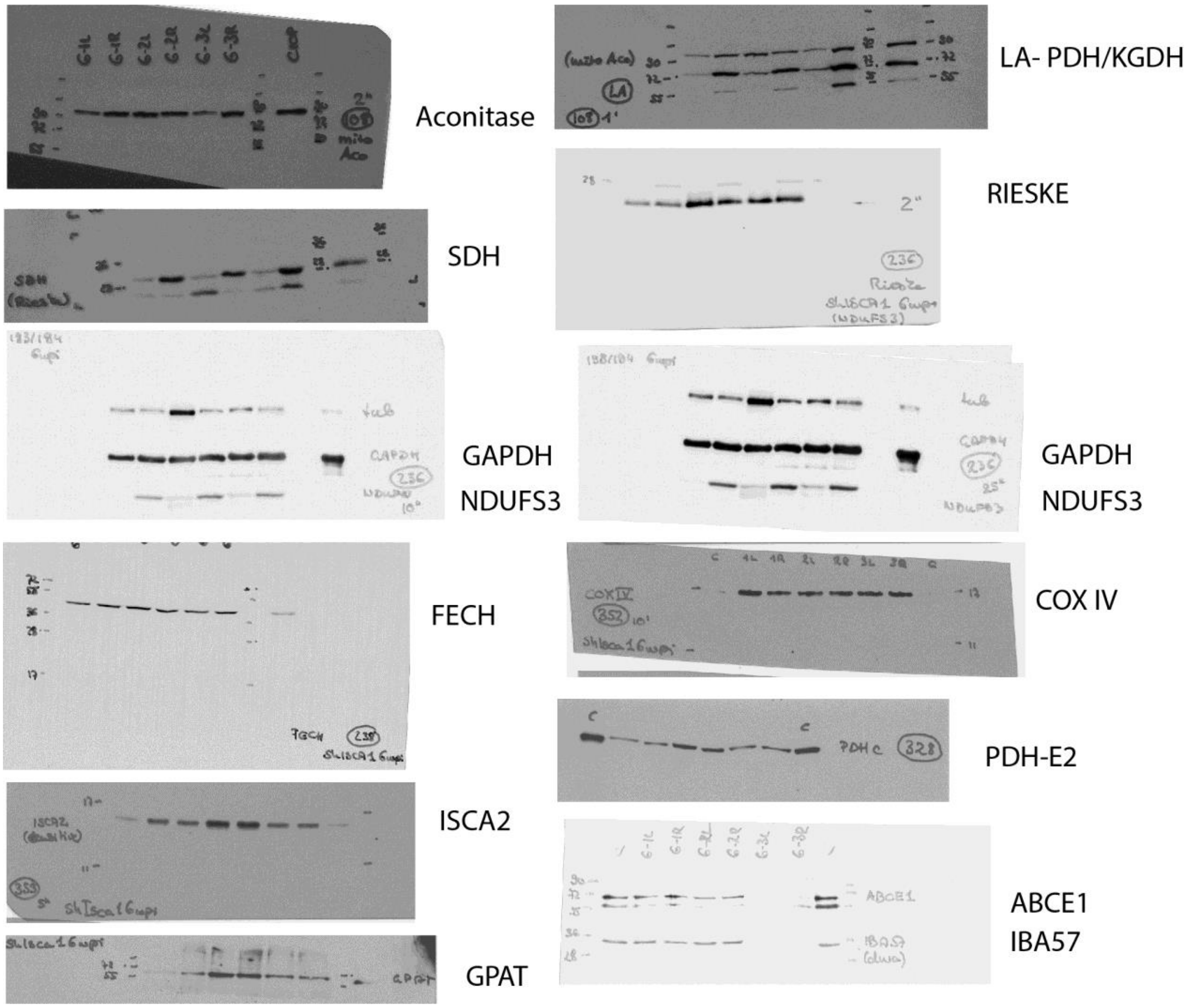

Original blots for Figure S2D:
ISCA2 knockdown 6 wpi

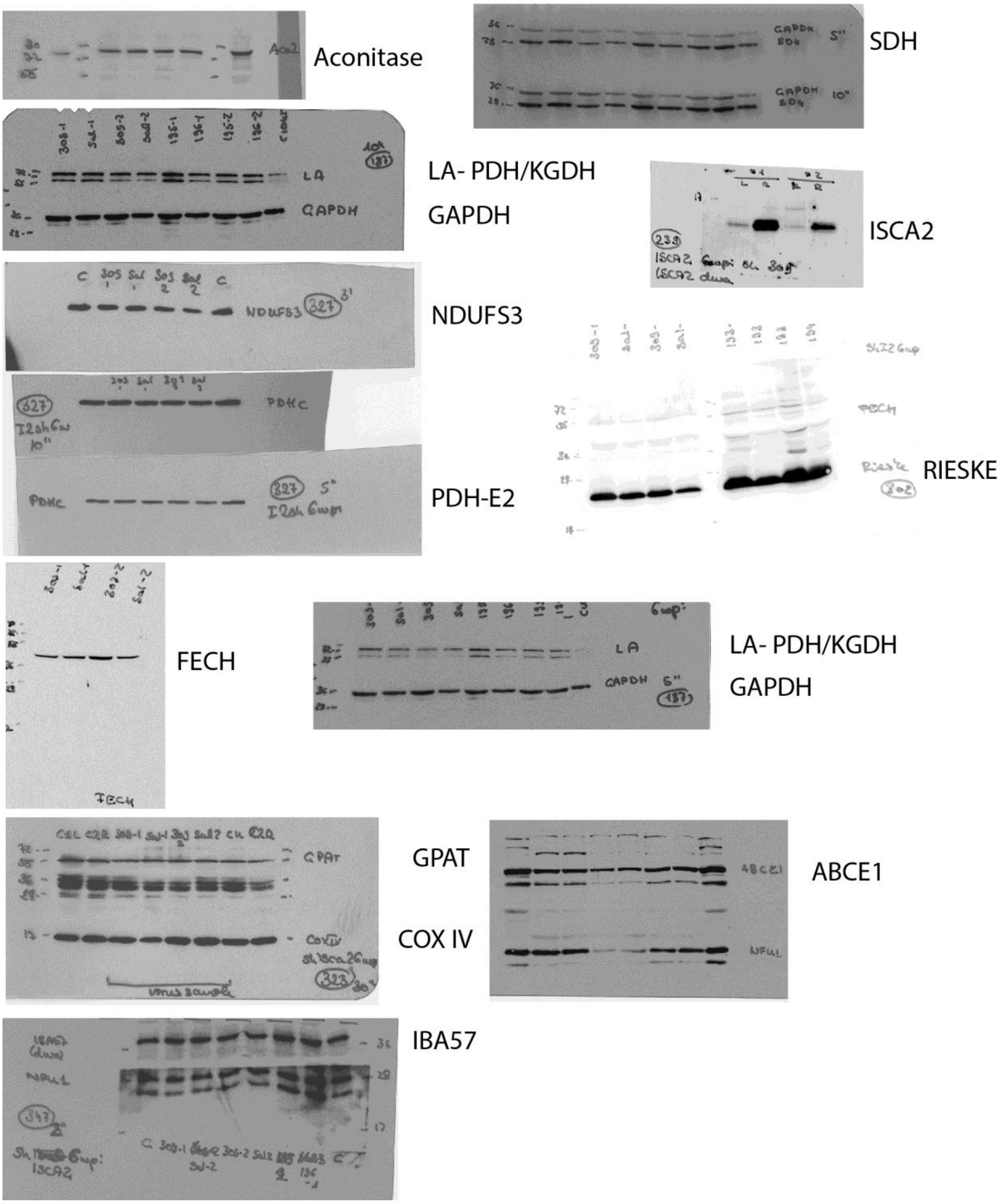

Original blots for Figure 5C and 5D:

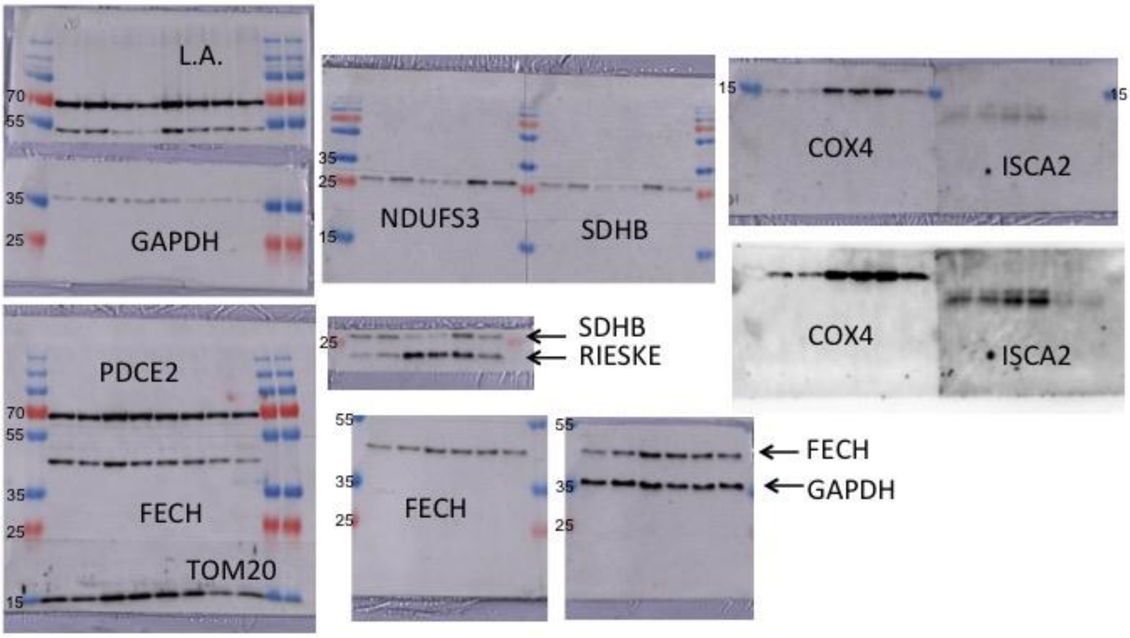

Original blots for Figure S2E:
IBA57 knockdown

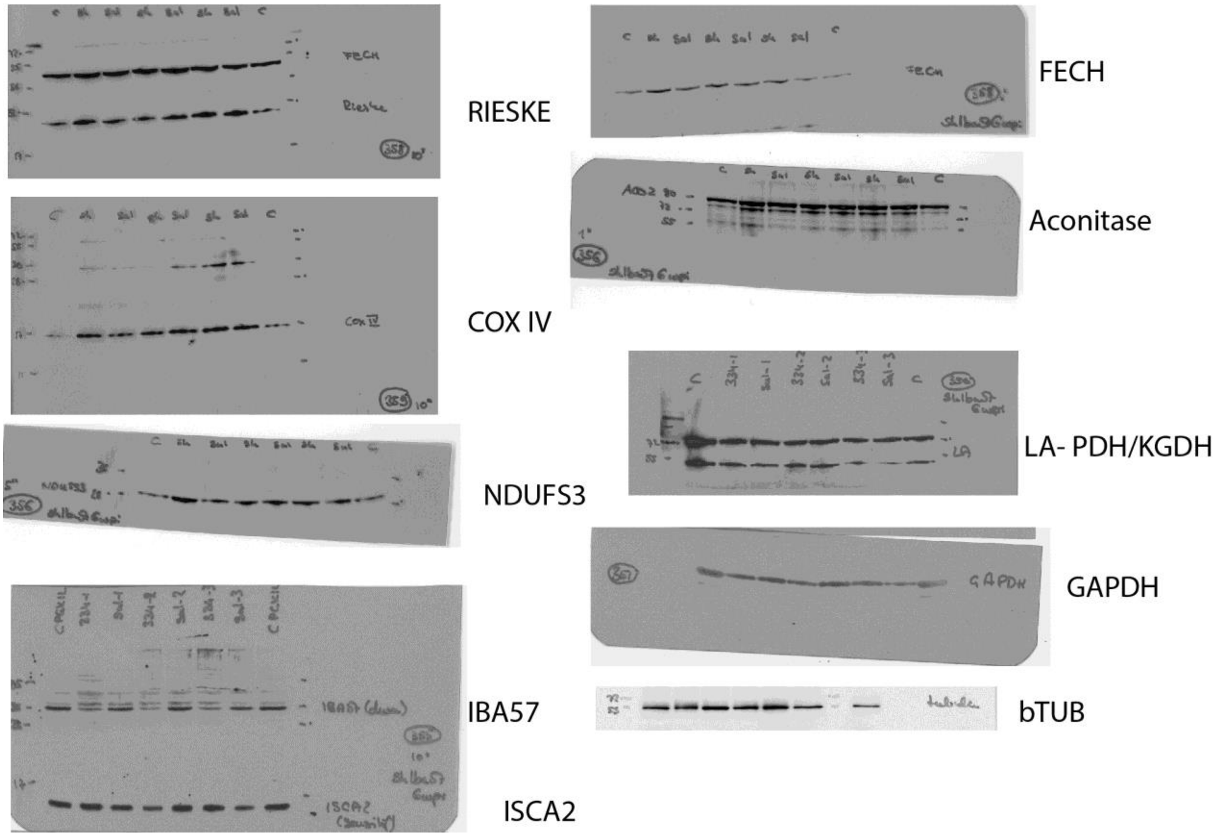

Supplementary Figure 4 Original blots for Figures 3, 4, 5 and S2

Supplementary Table 1 Values of the nuclear parameters and contributions for the six components issued from simulations of the spectra shown in Supplementary Figure 1C

	Species	δ (mm/s)	ΔE_Q (mm/s)	Γ (mm/s)[a]	% in control cell samples		% in induced ISCA cells	
ISCA2	HS Fe^{II}	1.31	2.88	0.55	22	*40*	22	*37*
	HS Fe^{II}	0.98	2.87	0.55	18		9	
	HS $Fe^{II}(Cys)_4$	0.69	3.17	0.34	0		6	
	$[Fe_4S_4]^{2+}$ & LS Fe^{II} heme	0.45	1.15	0.42	20	*60*	11	*34*
	Fe^{III} NP	0.48	0.61	0.56	40		23	
	$[Fe_2S_2]^{2+}$[b]	0.27<br>0.28	0.50<br>0.68	0.28<br>0.28	<2<br><2	*<4*	15<br>15	*30*

[a] The linewidth for HS Fe^{II} components were constrained to be equal. The same holds for the two sites of the $[Fe_2S_2]^{2+}$ clusters. [b] Isomer shift and quadrupole splitting values are those published for the $[Fe_2S_2]^{2+}$ cluster of ^{Nif}IscA and were fixed. The two sites were assumed to contribute in a 1:1 ratio.

Supplementary Table 2 Sequences of primers for PCR amplification, for mutagenesis and shRNA

GENE	PRIMERS	Used for
Isca1	FD: AATAATGGTACCGTGGACATGTCGGCGTCGTTGGTC<br>RV:<br>AATAATCTCGAGTCACTTATCGTCGTCATCCTTGTAATCCACGTGAAAGCTCTCACCGC	Flag-tagged protein
Isca1	FD: AATAATCATATGCATCATCATCATCATCACGTGAGCAAGAGAAAACTGCAG<br>RV: ATTATTGGATCCTCACACGTGAAAGCTCTCAC	His-tagged protein
Isca2	FD: AATAATGGTACCAGTAAGATGGCGGCCTCCAGGG<br>RV:<br>AATAATCTCGAGTCACTTATCGTCGTCATCCTTGTAATCGACTTTGACAGAGAAGGATG	Flag-tagged protein
Isca2	FD: AATAATCATATGCATCATCATCATCATCACACAACATCTTCCATTCCAGAGGC<br>RV: ATTATTGGATCCTCAGACTTTGACAGAGAAGGATG	His-tagged protein
Glrx5	FD: AATAATGAATTCGCGGGCATGAGCGCGTCCCTGAG<br>RV:<br>ATTATTCTCGAGTCACTTATCGTCGTCATCCTTGTAATCCTTGGAGTCTTGGTCCTTC	Flag-tagged protein
Iba57	FD: AATAATGAATTCCCCAAGATGGCAGCGGTGGCGC<br>RV:<br>ATTATTCTCGAGCTACTTATCGTCGTCATCCTTGTAATCCTTGGCAGCTGTGGGCCACC	Flag-tagged protein
Fdx2	FD: AATAATGAATTCGCTATCATGGCCGCCTCCATG<br>RV: ATTATTCTCGAGTCACTTATCGTCGTCATCCTTGTAATCGTGGGGCTTGGGGATGTG	Flag-tagged protein
shRNA-Isca1	GGAGATTCTGATGAAGAAGTTCAAGAGACTTCTTCATCAGAATCTCC	
shRNA-Isca2	GGTCATCCTTCTCTGTCAAAGTTCAAGAGACTTTGACAGAGAAGGATGACC	
shRNA-Iscu	GGCTCTACCACAAGAAGGTTGTTCAAGAGACAACCTTCTTGTGGTAGAGCC	
shRNA-Iba57	GCGTATGCCCATTTCCTGAATTTCAAGAGAATTCAGGAAATGGGCATACGC	
shRNA-scrambled	GGAGGGTACGATAAATTGATTCAAGAGATCAATTTATCGTACCCTCC	
Isca1	FD: GTACACAAAGACAAAAGGAGACAGTGATGAAGAAGTTATTCAAG<br>RV: CTTGAATAACTTCTTCATCACTGTCTCCTTTTGTCTTTGTGTAC	Mutagenesis for ISCA1^{R}

Supplementary Table 3 List of Antibodies

**Antibody against**	**details**	**Provider**	**Dilution**
ISCA2	Home made rabbit polyclonal (R2883)	IGBMC	1: 2,000
IBA57	Home made rabbit polyclonal (R3230)	IGBMC	1: 1,000
NFU1	Rabbit polyclonal against GST-NFU1	kindly provided by T. Rouault, NIH, Bethesda, USA	1: 5,000
GLRX5	Rabbit polyclonal against peptide YLDDPELRQGIKDYS	kindly provided by C. Bouton, ICSN, Gif sur Yvette, France	1: 1,000
ISCU	Home made rabbit polyclonal (R2386)	IGBMC	1: 1,000
FLAG	Mouse monoclonal M2 antibody, F3165	SIGMA	1: 5,000
Lipoic acid (LA)	Rabbit polyclonal, 437695	Calbiochem	1: 5,000
SDHB (complex II)	Mouse monoclonal, 21A11AE7	Invitrogen	1: 5,000
NDUFS3 (complex I)	Mouse monoclonal, 439200	Invitrogen	1: 5,000
Rieske, UQCRFS1 (complex III)	Mouse monoclonal, ab14746	Abcam	1: 5,000
COX IV (complex IV)	Mouse monoclonal, ab14744	Abcam	1: 1,000
GPAT	Home made rabbit polyclonal (2374)	IGBMC	1: 1,000
FECH	Home made rabbit polyclonal (2381)	IGBMC	1: 1,000
ACO2	Home made rabbit polyclonal (2377)	IGBMC	1: 10,000
GAPDH	Mouse monoclonal, MAB374	Millipore	1: 40,000
β-Tubulin	Home made mouse monoclonal, 1TUB-2A2	IGBMC	1: 20,000-1:40,000
ABCE1	Rabbit polyclonal, ab32270	Abcam	1: 1,000
NDUFS5 (complex I)	Mouse monoclonal, ab188510	Abcam	1: 1,000
NDUFS6 (complex I)	Mouse monoclonal, ab156099	Abcam	1: 500

Supplementary Table 4 Sequences of primers for qRT-PCR

**Genes**	**primers**
Isca1 ORF Fd	CTGAGCATGTGGGTCTGAAA
Isca1 ORF Rv	TTCCCTTGATGTTGGGGTTA
Isca1 UTR Fd	CAAGCCAATGTGGGAAGAGT
Isca1 UTR Rv	CAAAGCAGGCTACAGCACAA
Hprt Fd	GTAATGATCAGTCAACGGGGGAC
Hprt Rv	CCAGCAAGCTTGCAACCTTAACCA
